# Identification of antagonistic activity against *Fusarium*, and liquid fermentation of biocontrol *Bacillus* isolated from wolfberry (*Lycium barbarum*) rhizosphere soil

**DOI:** 10.3389/fmicb.2025.1601945

**Published:** 2025-07-15

**Authors:** Na Xi, Teng-Da Liu, Yu-Zhou Zhang, Kuo Liu, Hua-Ying Du, Pei-Wen Gu, Ze-Yang Yu, Yu-Yang Song

**Affiliations:** ^1^School of Agriculture, Ningxia University, Yinchuan, Ningxia, China; ^2^Ningxia Rural Science and Technology Development Center, Yinchuan, Ningxia, China; ^3^College of Enology, Northwest Agriculture and Forestry University, Yangling, Shanxi, China; ^4^Ningxia Forest Pest Control and Quarantine Station, Yinchuan, Ningxia, China

**Keywords:** *Bacillus*, isolation, wolfberry root rot, antagonistic effects, fermentation optimization

## Abstract

**Introduction:**

*Fusarium* is a kind of plant pathogenic fungus that is widely present in the soil. It can trigger a variety of plant diseases, such as wilt and root rot, which pose a serious threat to agricultural production.

**Methods:**

In this study, a strain of *Bacillus subtilis* was identified by morphology, physiology, biochemistry, and molecular biology and was named LK−1.

**Results:**

The antagonistic curve showed that the best antagonistic fermentation time was 48 h. The fermentation broth and bacterial suspension of LK−1 had sound antagonistic effects on the mycelium or spores of *F. oxysporum*; when the mycelium or spores of *F. oxysporum* were treated with LK−1, they grew abnormally. Compared to fermentation broth, the antagonistic effect of sterile filtrate was lower. In addition, compared to the *Lycium barbarum* seedlings in the control group, the plant height, root length, aboveground fresh weight, underground fresh weight, aboveground dry weight, underground dry weight, and leaf area indexes of *L. barbarum* seedlings treated with LK−1 fermentation broth (1 × 10^8^ cfu/mL) were all significantly higher. Using the one-way test, Plackett–Burman test, and Box–Behnken test, the fermentation medium and conditions for strain LK−1 were ultimately determined to be 0.5% glucose, 2% beef extract, 2% NaCl, and 0.5% yeast powder, with a fermentation time of 46 h, an inoculum size of 1%, a pH of 6.6, and a rotational speed of 170 rpm. Validation experiments showed that the bacterial inhibition rate under these conditions reached 62.5%, which was 1.43% higher than the theoretical value and 8.06% higher than the unoptimized value (54.44%).

**Discussion:**

LK-1 had sound preventive and control effects against many kinds of *Fusarium*.

## 1 Introduction

*Lycium barbarum*, a deciduous shrub belonging to the *Solanaceae* family, is a traditional Chinese medicine with a long history. It is renowned worldwide for its outstanding nutritional and medicinal values (Xie et al., 2023). The nutritional profile of the *L. barbarum* berry includes polysaccharides (Zhou et al., 2018; Tian et al., 2019), minerals (Kulaitienė et al., 2020), vitamins (Donno et al., 2015), carotenoids (Inbaraj et al., 2008), flavonoids (Zhou et al., 2017), a substantial number of free amino acids (Dandan et al., 2020), and other metabolites. Much medical research has demonstrated that *L. barbarum* berries possess a multitude of beneficial properties, including anti-aging properties (Zhu et al., 2022), antioxidant properties (Yang et al., 2022), antitumor properties (Qin et al., 2022), immunomodulatory properties (Zhu et al., 2020), neuroprotective properties (Hu et al., 2018), and being good for the reproductive system (Tang et al., 2017), which makes wolfberry a well-selling product.

Due to its high economic value, salinity tolerance, and drought tolerance (Wei et al., 2006), *L. barbarum* cultivation in the dry areas of northwest China has increased dramatically, improving local economic returns. However, root rot disease seriously affects the yield and quality of *L. barbarum* and severely restricts local economic development.

*Fusarium* has been identified as the major cause of root rot, and its host range is extensive, which makes it one of the most problematic plant pathogens worldwide (Estrada Jr et al., 2010; Maryani et al., 2019). Root rot in *L. barbarum* caused by *Fusarium culmorum* and *Fusarium. equiseti* was first reported in Qinghai, China (Bai et al., 2020). The presence of *Fusarium* spp., including *F. oxysporum*, *F. solani*, *F. tricinctum*, *F. chlamydosporum,* and *Alternaria alternata*, which are responsible for root rot disease in wolfberry plants in China, was confirmed (Uwaremwe et al., 2021). *Fusarium oxysporum* was the dominant species of wolfberry root rot in Ningxia. With the increasing emphasis on food safety and the need for sustainable control of plant disease, governments and plant protection experts have emphasized biological control. *Bacillus* is a beneficial plant growth-promoting bacterium with the advantages of being environmentally friendly, protecting plants from disease-causing organisms, and not generating pesticide resistance (Allard-Massicotte et al., 2016; Donato et al., 2017), and is one of the most optimal potential strategies for controlling soil-borne diseases (Jiang et al., 2015). It has been reported that a variety of *Bacillus* species, such as *B. subtilis* (Jan et al., 2023), *B. cereus* (Aydi Ben Abdallah et al., 2016), *B. amyloliquefaciens* (Wan et al., 2018), and *B. velezensis* (Jiang et al., 2019), can protect against the disease by mechanisms such as competitive action (Beauregard et al., 2013; Tan et al., 2016), antagonism (Caulier et al., 2019), induced systemic resistance (Yan et al., 2002; Pieterse et al., 2014), and the promotion of plant growth (Patel et al., 2023).

Plant growth promotion and resistance induction are two significant factors determining *Bacillus*’s value in agricultural applications (Kamil et al., 2018; Zalila-Kolsi et al., 2022). Recently, the production of *Bacillus* metabolites has been enhanced through fermentation engineering, thereby increasing crop yield and preventing disease. Many factors affect the microbial fermentation process (Tang et al., 2004; Liu et al., 2007; Dhandhukia and Thakkar, 2008), which include two main aspects as follows: first, the optimization of medium composition; second, the optimization of fermentation conditions. Statistical methods using screening and response surface methodology or artificial neural networks offer several advantages over conventional methods in optimizing numerous multi-factorial processes or formulations (Aguirre and Bassi, 2013; Samavati, 2013; Bhatia et al., 2014; Samaram et al., 2015; Hsieh et al., 2016). The main advantage of response surface methodology is that it reduces the number of trials needed to evaluate multiple variables and their interactions (Zhong et al., 2012). In this study, *Bacillus* was isolated and characterized in the Ningxia region, and its biocontrol effect on root rot of *L. barbarum* was determined. Its biocontrol ability against the root rot of *L. barbarum* was improved by response surface methodology.

## 2 Materials and methods

### 2.1 Materials

Soil samples were collected from Ningxia Nanliang No. 2 Farm (30 copies), the Chinese wolfberry nursery of the Academy of Agricultural Sciences (20 copies), and the Saishangjiangnan Farm in Ningxia (25 copies). Surface soil was removed, and soil samples were randomly taken from a depth of 5––15 cm around the root system of *L. barbarum*.

Pathogens, namely, *F. oxysporum, F. equiseti, F. acuminatum, F. avenaceum, F. solani, F. tricinctum,* and *F. chlamydosporum* were preserved in the Department of Plant Protection laboratory, Faculty of Agriculture, Ningxia University, China.

Test materials include Luria–Bertani medium (LB medium), Potato Dextrose Agar medium (PDA medium), Congo red, methyl red, Voges–Proskauer medium, indole medium, starch hydrolysis medium, sugar fermentation, and gelatin liquefaction medium specified in “Bergey’s Manual of Systematic Bacteriology” (Senesi et al., 2001).

### 2.2 Isolation and identification of antagonistic bacteria

#### 2.2.1 Isolation and screening of antagonistic bacteria

Soil samples were diluted to three concentrations (10^−3^, 10^−4^, and 10^−5^, w/v) with sterile water. A volume of 20 microliters of soil suspension was inoculated onto a Luria–Bertani agar medium (LB medium) and incubated for 24 h at a temperature of 27°C. Individual colonies with different morphological characteristics were picked and inoculated on LB medium; subsequently, the sample was isolated and purified through growth at 28°C for 24 h (Wang et al., 2020). Purified strains were stored at 4°C for backup. The plate standoff method (Naveed et al., 2022) was then performed, and *F. oxysporum* was used as an indicator. *Fusarium oxysporum* was inoculated in the center of the PDA medium, and *Bacillus* was inoculated equidistant around *F. oxysporum*, while only *F. oxysporum* was inoculated in the control group and cultured at 27°C for 5 days. The diameter of pathogen colonies was measured using the crossover method to screen for strains with biocontrol effects against *F. oxysporum*.


$$\text{Inhibition Rate}\;(\%)=(\text{diameter}-{\text{diameter}}_{\text{treatment}})/d_{\mathrm{CK}}\times 100\%.$$

Where “d” represents the diameter of the colony, and the subscript “CK” indicates the control fungus (*F. oxysporum*).

#### 2.2.2 Morphological, physiological, and biochemical characterization

The morphological, physiological, and biochemical characteristics of the isolated and purified strains were identified using sugar fermentation experiments, indole experiments, Voges–Proskauer experiments, methyl red experiments, Congo red experiments, starch hydrolysis experiments, and contact enzyme activity tests specified in the Manual of Systematic Identification of Common Bacteria (Dong and Cai, 2001).

#### 2.2.3 Molecular identification

The total DNA of the purified strain was extracted and purified using a bacterial DNA kit (Tian Gen., Beijing, China). The primers used were 16S (27F: AGAGTTTGATCMTGGCTCAG, 1492R: GGCTACCTTGTTACGACTT), *gyrB* (F: AACGATTTTGCGGGCTGAG, R: GCGTAAACATCACGATTGAAGAC), *rpoB* (F: AGCCGTTATTTGTAAACACCCTG, R: CGTCGTTGACTTCCGCAC), and *recA:* (F: TCACGCAATCGCTGAGGTT, R: CAAGACGCACGGAAGAATAGAAT). The PCR system consisted of 12.5 μL of the 2 × San Taq PCR Mix, 1 μL of the DNA template, 1 μL of each primer, and 9.5 μL of ddH_2_O. The PCR conditions were pre-denaturation at 94°C for 2 min, denaturation at 94°C for 30 s, annealing at 56°C for 1 min, and extension at 72°C for 1 min, and the total PCR system included 35 cycles and finally extension at 72°C for 10 min. Next, 1% agarose gel electrophoresis was performed, and gel images were obtained using a gel imaging system. The amplified 16S rRNA PCR products were sequenced (all purchased from Shanghai Sangon Biotech, Shanghai, China). After obtaining the sequences, the NCBI BLAST program was used to compare the 16S rRNA sequences with those in the database, and a phylogenetic tree was constructed using the maximum likelihood (ML) method with 1,000 bootstrap replications in MEGA 7.0 software (Sharma and Kumar, 2024).

### 2.3 Defensive role

#### 2.3.1 Antagonistic ability evaluation of five *Bacillus* strains

Employing the plate confrontation method, filter paper disks infused with *Bacillus* fermentation liquid and each of 11 pathogenic fungi were inoculated onto PDA medium at a temperature of 27°C for a duration of 5 days; the control group was solely inoculated with the pathogenic fungus. The experiment was conducted with three independent replicates. The diameters of the colonies were measured using the criss-cross method, and the inhibition rates were calculated to identify the optimal antagonistic bacteria.

#### 2.3.2 Different fermentation time evaluation of *Bacillus subtilis* LK−1

The activated *B. subtilis* LK−1 was inoculated into 50 mL triangular flasks containing 20 mL of LB medium and incubated at 37°C with a shaking speed of 180 rpm. The bacterial inhibition rate of the culture medium was assessed at various time intervals.

#### 2.3.3 Influence of *Bacillus subtilis* LK−1 on the mycelium and spores of *Fusarium oxysporum*

Employing the flat plate confrontation method, the *F. oxysporum* was inoculated in the central region of a PDA culture medium on a flat plate. Three inoculants (fermentation solution, sterile filtrate, and bacterial suspension) were inoculated equidistantly around *F. oxysporum*. Each treatment was conducted in triplicate. Following a 5-day incubation period at 27°C, the colony diameter was measured, and the morphological alterations of the hyphae at the interface between diseased and healthy tissues were examined.

#### 2.3.4 Effect of *Bacillus subtilis* LK−1 on the spores of *Fusarium oxysporum*

An equal volume of *F. oxysporum* (spore suspension) and *B. subtilis* LK−1 (fermentation liquid) were combined. The spore germination rate, the inhibition rate of spore germination, and the morphological changes in spores were assessed at 2, 4, 6, 8, 10, and 12 h after combining. In the control, the spore suspension of *F. oxysporum* was replaced with sterile water.

#### 2.3.5 The growth-promoting effect of *Bacillus subtilis* LK−1 on *Lycium barbarum*

Once the *L. barbarum* seedlings developed two true leaves, the fermentation liquid of the biocontrol bacteria and the spore suspension of *F. oxysporum* were irrigated to the root zone as follows: (1) inoculation with 40 mL of water, set as the control group (CK); (2) inoculation with 20 mL of LK−1 and 20 mL of water (E); (3) inoculation with 20 mL of LK−1 and 20 mL of *F. oxysporum* (E + P); and (4) inoculation with 20 mL of water and 20 mL of *F. oxysporum* (P). After 30 days, various physical indexes of *Lycium* plants were measured, including plant height, root length, leaf number, leaf length, leaf width, and leaf area, as well as fresh and dry weights of both the aboveground and belowground parts.

### 2.4 Growth curves

The activated strain LK−1 was inoculated into LB medium and incubated at 37°C with a shaking speed of 180 rpm, while OD_600_ values were measured at various time intervals.

The growth curve was plotted with time as the horizontal coordinate and OD_600_ as the vertical coordinate. The cultured solution where LK−1 grows quickly and at the log phase in the growth curve will be chosen as the seed solution.

### 2.5 Single-factor test

In the basal fermentation medium with an initial pH of 7, different inorganic salts were considered in KCl₂, MgSO₄, ZnSO₄, Ca₃(PO₄)₂, and NaCl, and different concentrations of inorganic salts were considered in 0.5, 1.0, 1.5, 2.0, and 2.5% (m/v, g/mL). A 2% seed solution was inoculated and cultured at 37°C and 180 rpm for 48 h, and the effect of the cultured solution on antagonism was determined using the plate confrontation method with three replicates.

The optimal fermentation conditions identified previously served as a baseline, with the carbon source concentration maintained as specified. In the basal medium with an initial pH of 7, the carbon sources were substituted with corn flour, soluble starch, maltose, fructose, and glucose. The effect of the cultured solution on antagonism was evaluated in the same way as mentioned above.

The optimal fermentation conditions identified previously served as a baseline with an initial pH of 7 and carbon source concentrations maintained as specified. The two nitrogen sources in the basal medium were, respectively, substituted with beef extract, NaNO_3_, KNO_3_, (NH_4_)_2_SO_4_, and peptone/yeast powder. The effect of the cultured solution on antagonism was evaluated in the same way as mentioned above.

Based on the optimal fermentation conditions screened earlier with an initial pH of 7, the fermentation times were set as 12 h, 24 h, 36 h, 48 h, 60 h, and 72 h. A 2% seed solution was inoculated and cultured at 37°C and 180 rpm, and the effect on antagonism was determined using the plate confrontation method with three replicates.

Based on the optimal fermentation conditions screened earlier with an initial pH of 7, the initial inoculum was set as 1.0, 2.0, 3.0, 4.0, 5.0, and 6.0%. The inoculated medium was cultured at 37°C and 180 rpm for 36 h (evaluated earlier), and the effect on antagonism was determined using the plate confrontation method with three replicates.

Based on the optimal fermentation conditions screened earlier, the initial pH was set as 3, 5, 7, 9, 11, and 13. A 1% seed solution was inoculated and cultured at 37°C and 180 rpm for 36 h, and the effect of the cultured solution on antagonism was determined using the plate confrontation method with three replicates.

Based on the optimal fermentation conditions investigated earlier, the rotational speeds were set at 100 rpm, 120 rpm, 140 rpm, 160 rpm, 180 rpm, and 200 rpm, a 1% seed solution was inoculated and cultured at 37°C for 36 h, and the effect of the cultured solution on antagonism was determined using the plate confrontation method with three replicates.

### 2.6 Response surface optimization

#### 2.6.1 Plackett–Burman experiment

Based on the one-way test, Design-Expert 12 software was used to design the PB test protocol to screen the main influencing factors from seven influencing factors using the inhibition rate (Y) as the response value (Table 1).

**Table 1:** Factors and levels of Plackett–Burman experiments.

PB experimental factors	−1	1
X1 Glucose (%)	0.5	2.5
X2 NaCl (%)	1	2
X3 Beef Paste (%)	1	2
X4 Time (h)	12	36
X5 Inoculum (%)	1	3
X6 pH	3	7
X7 Rpm	100	180

#### 2.6.2 Steepest climb test

The steepest climb test was performed based on the analysis of the PB test results to determine the center of the response surface optimization.

#### 2.6.3 Box–Behnken tests

Based on the variables screened in the PB test and the steepest climb test, the response values were analyzed using regression equations, with the inhibition rate (Y) designated as the response variable. This analysis was conducted using Design-Expert 12 software to design a three-factor, three-level experiment aimed at determining the optimal fermentation conditions for strain LK−1.

## 3 Results

### 3.1 Isolation and screening of antagonistic bacteria

#### 3.1.1 Isolation of antagonistic bacteria and determination of inhibition rate

**Figure 1:**
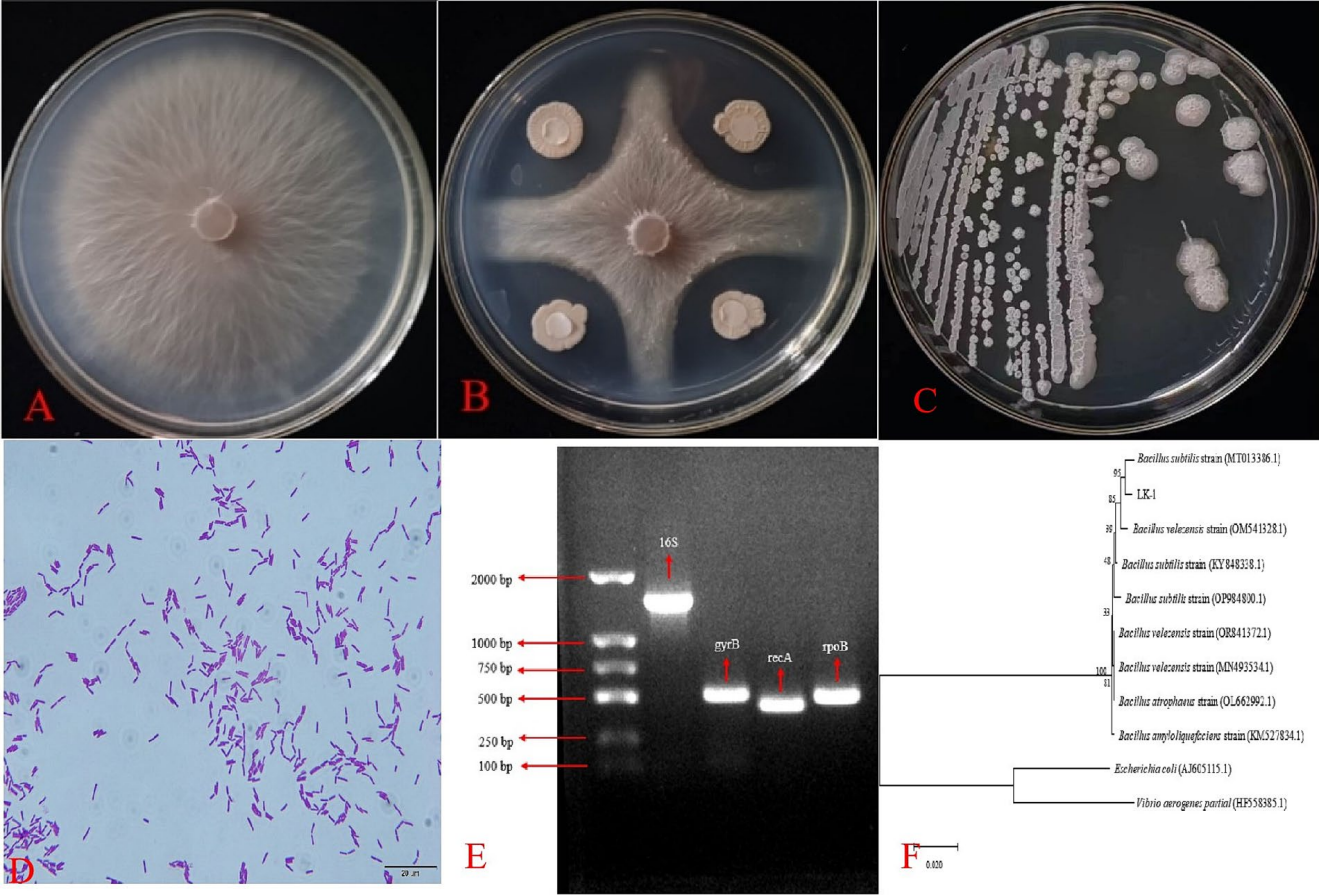
Biocontrol effect, morphological characteristics, and molecular biology identification of *Bacillus.* **(A)** *Fusarium oxysporum.* **(B)** Confrontation assay between *F. oxysporum* and *Bacillus.* **(C)** Macroscopic morphology. **(D)** Microscopic morphology. Electrophoresis **(E)** and phylogenetic tree **(F)** of strain LK-1.

Seventy-five soil samples were collected from various regions in Ningxia, from which 136 bacterial strains exhibiting diverse morphologies were isolated. The *F. oxysporum* was set as the indicator organism for the chosen antagonistic bacteria, and five strains that demonstrated antagonistic activity against *F. oxysporum* were identified. The inhibition rate of strain LK−1 reached 58.48%, the inhibition rate of strain LK-2 was 53.91%, that of LK-3 was 56.52%, that of LK-4 was 57.39%, while that of LK-5 was the lowest, at 44.13%. Among these, LK−1 showed the best pathogen control ability with a pathogen inhibition rate of 58.48% and has been preserved for further study (Figures 1A,B).

#### 3.1.2 Morphological, physiological, and biochemical characterization and molecular biology identification

LK−1 exhibited a positive Gram stain reaction and displayed a rod-shaped morphology. The individual colonies cultured on LB medium were characterized by their white coloration and wrinkled margins (Figures 1C,D).

The physiological and biochemical results were referred to “Bergey’s Manual of Systematic Bacteriology” and “Manual of Systematic Identification of Common Bacteria,” and combined with the morphological characteristics, the five strains were initially identified as *Bacillus* spp. (Table 2).

**Table 2:** Identification of physiological and biochemical characteristics of biocontrol strain LK-1.

Physiological and biochemical indicators/strains	LK-1	LK-2	LK-3	LK-4	LK-5
Glucose fermentation	+	+	+	−	+
Sugar fermentation	+	+	+	−	+
Fructose fermentation	+	−	+	−	+
Xylose fermentation	−	−	−	−	−
Congo red assay	−	−	−	−	−
Methyl red test	−	−	−	+	+
Voges–Proskauer experiments	+	+	+	+	+
Indole experiment	−	−	−	−	−
Starch hydrolysis experiment	+	−	−	−	+
Exposure enzyme assay	+	+	+	+	+
Gelatin liquefaction experiment	+	+	+	+	+

“+” denotes positive; “−” denotes negative.

#### 3.1.3 Molecular biology identification

The sequence of strain LK−1 was amplified by PCR using the bacterial universal primer 16S rRNA, specific primers *gyrB*, *rpoB*, and *recA*, and strain LK−1 DNA as a template. The fragments amplified from strain LK−1 were all around the expected amplified bands (Figure 1E).

The 16S PCR product was sent to Shanghai Sangon Biologic Engineering Co., Ltd. for sequencing, and a sequence of 1,253 bp was obtained and uploaded to NCBI under accession number PP582380.

The sequences obtained from sequencing were subjected to BLAST homologous sequence comparison in NCBI. In total, 10 strains with high homology to the target sequences were selected, and a phylogenetic tree was constructed using MEGA 7.0 software (Figure 1F). Combined with morphological, physiological, and biochemical characteristics, strain LK−1 was identified as *B. subtilis*.

### 3.2 Defensive role

#### 3.2.1 Screening for quality antagonistic bacteria

Co-infection with various *Fusarium* species often exacerbates the root rot disease affecting *L. barbarum*. Therefore, selecting a strain of antagonistic bacteria that exhibits solid inhibitory effects against multiple *Fusarium* strains is essential. The biocontrol effect of strain LK−1 was significantly higher than that of the other four strains, especially against the *F. oxysporum* and *F. chlamydosporum* of Ningxia. Consequently, the selection of the antagonist bacterium LK−1 for future experiments is warranted (Table 3; Supplementary Figure 1).

**Table 3:** Screening of high-quality antagonistic bacteria.

Pathogenic fungi/antagonistic bacteria (%)	LK-1	LK-2	LK-3	LK-4	LK-5
A. *F. equiseti*	45.64 ± 1.74a	44.97 ± 0.58a	43.29 ± 2.32ab	38.59 ± 3.63b	31.21 ± 3.81c
B. *F. oxysporum*	57.07 ± 1.20a	49.74 ± 0.79b	49.74 ± 0.79b	35.34 ± 2.52c	37.7 ± 0.91c
C. *F. oxysporum*	36.61 ± 1.55a	40.34 ± 1.55a	37.29 ± 1.55a	28.47 ± 1.55b	15.59 ± 4.66c
D. *F. acuminatum*	42.71 ± 1.17a	41.69 ± 0.59a	41.02 ± 1.02a	24.07 ± 3.11b	16.95 ± 1.55c
E. *F. oxysporum*	50 ± 0.71a	48.57 ± 0.71a	50.48 ± 1.09a	39.29 ± 1.89b	42.86 ± 0c
F. *F. avenaceum*	52.97 ± 0.45a	52.2 ± 2.24a	48.32 ± 1.18b	42.89 ± 0.45c	41.09 ± 1.55c
G. *F. solani*	49.62 ± 0.88a	51.14 ± 0.44a	51.39 ± 0.76a	45.32 ± 1.52b	47.09 ± 0.88b
H. *F. tricinctum*	55.94 ± 3.74a	58.91 ± 0.43a	56.93 ± 1.49a	46.53 ± 1.49b	42.08 ± 4.52b
I. *F. equiseti*	46.56 ± 0.57a	43.93 ± 1.70ab	40.33 ± 5.05bc	35.41 ± 1.50 cd	29.51 ± 3.72d
J. *F. acuminatum*	50.76 ± 0a	48.33 ± 0.53b	45.9 ± 0.53c	36.17 ± 0d	25.23 ± 0.91e
K. *F. chlamydosporum*	57.37 ± 0.77a	56.03 ± 0.77a	56.25 ± 0.77a	40.63 ± 0.77b	43.75 ± 1.77c

A, C, and D were isolated from *Astragalus*, B was isolated from *Lycium barbarum*, and E–K were isolated from alfalfa. The values in the table are the mean ± standard errors, and different letters in the peer group indicate the significance of the difference at the 0.05 level.

#### 3.2.2 Screening for optimal antagonistic effect

**Figure 2:**
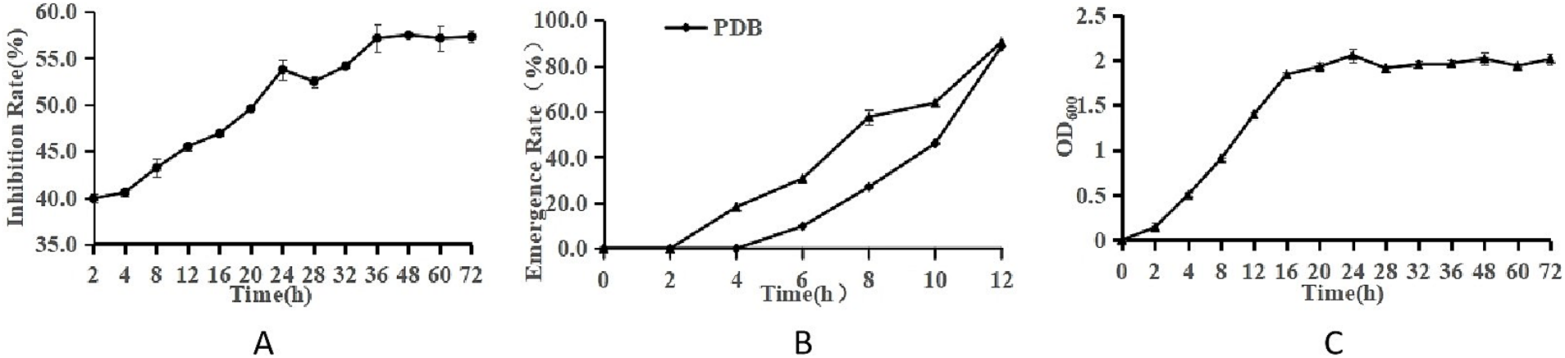
Growth characteristics of *Bacillus* and *F. oxysporum.* **(A)** Curve of antagonism. **(B)** Spore germination rate. **(C)** Curve of growth. The ordinate of panels **A–C** is the antagonistic effect, the spore germination rate, and OD_600_, respectively. The abscissa is culture time.

Figure 2A presents the inhibitory effect of LK−1 on *Fusarium*, where the inhibition rate increases over time, peaking at 48 h (Figure 2A).

Figure 2B compares the emergence or growth of microorganisms under sterile water treatment conditions. The emergence rate under PDB treatment is significantly higher than that under sterile deionized water treatment, indicating that PDB may promote the emergence or growth of microorganisms.

Figure 2C presents a typical growth curve of LK−1, where the increase in OD value reflects the increase in the number of microorganisms. The peak at 24 h and eventual stabilization likely indicate that the microorganisms have reached their maximum growth density. LK−1 exhibits logarithmic growth during the initial 0–24 h. Following this period, it transitions into a stabilization phase. During the logarithmic growth phase, this bacterium demonstrates rapid proliferation and heightened metabolic activity, rendering it particularly suitable for use as a seed fluid.

The most pronounced antagonistic effect was observed in the fermentation broth after 48 h of incubation (Figure 2), following which a slight decrease in this effect occurred, likely attributable to cell lysis. Subsequent experiments were conducted using the fermentation broth of strain LK−1, which had been cultivated for 48 h.

#### 3.2.3 Effect of *Bacillus subtilis* on the mycelium

The *B. subtilis* solution exhibited the highest mycelial inhibition rate of 54.44%, followed by the bacterial suspension with a secondary inhibition rate. At the same time, the sterile filtrate demonstrated the lowest inhibition efficacy (Table 4).

**Table 4:** Antagonistic effects of *Bacillus subtilis* LK-1 on spores and mycelia.

Deal with	Spore germination (%)	Spore germination inhibition (%)	Inhibition rate (%)
Fermentation broth	25.82 ± 7.3a	71.42 ± 8.08a	54.44 ± 0.77a
Sterile filtrate	77.01 ± 4.84b	14.74 ± 5.36b	18.44 ± 0.38b
Bacterial suspension	19.44 ± 4.74a	78.48 ± 5.24a	53.78 ± 1.39a
Sterile water	90.33 ± 3.29c	0	7.50 ± 0.10c

The lowercase letters represent the significant differences in spore germination rate, spore germination inhibition rate, and inhibition rate between different treatments of LK-1.

**Figure 3:**
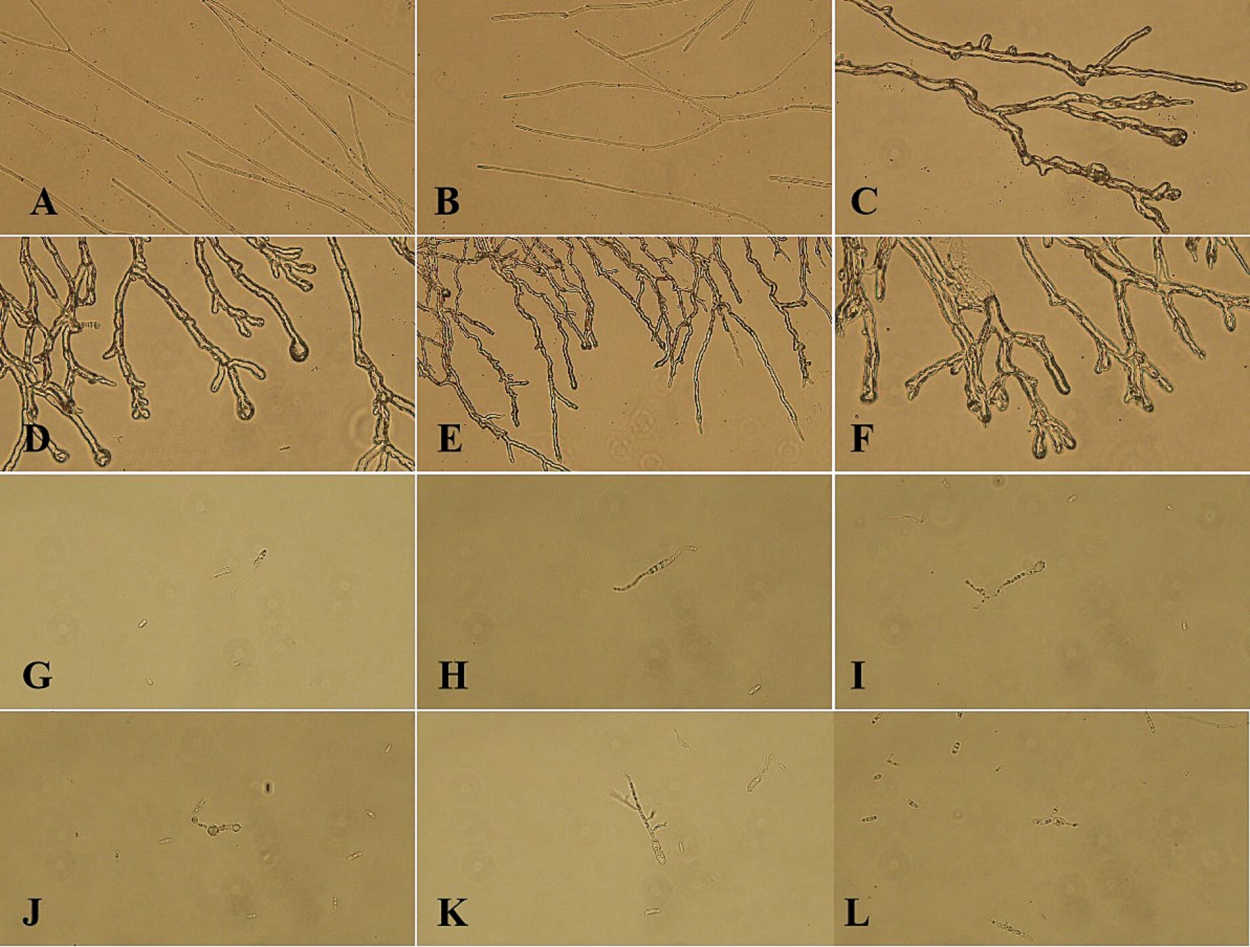
Morphological changes in mycelium and spores of the biocontrol strain LK-1. **(A)** Under normal culture conditions, the mycelium exhibits a consistent morphology characterized by uniform thickness and length. **(B)** Under normal culture conditions, the mycelium exhibits a consistent morphology characterized by uniform thickness and length. **(C)** Mycelium subjected to LK-1 treatment demonstrates abnormal branching. **(D)** Mycelium subjected to LK-1 treatment demonstrates expansion and abnormal branching. **(E)** Mycelium subjected to LK-1 treatment demonstrates abnormal branching. **(F)** Mycelium subjected to LK-1 treatment demonstrates abnormal branching and lysogenic effects. **(G)** Spores appear as elongated germ tubes. **(H)** Spores appear as elongated germ tubes. **(I)** Following treatment with strain LK-1, the spores appear as deformation. **(J)** Following treatment with strain LK-1, the spores appear as inflation. **(K)** Following treatment with strain LK-1, the spores appear as deformation. **(L)** Following treatment with strain LK-1, the spores appear as deformation.

Under normal culture conditions, the mycelium exhibits a consistent morphology characterized by uniform thickness and length, as well as straight growth patterns (Figures 3A,B). In contrast, mycelium subjected to LK−1 treatment demonstrates abnormal branching (Figures 3C–F), expansion (Figure 3D), and even lysogenic effects (Figure 3F). These alterations lead to pathological phenomena such as cell membrane rupture and leakage of intracellular inclusions.

#### 3.2.4 Effect of *Bacillus subtilis* on the spores of pathogenic fungi

The germination rate of spores cultured in sterile water consistently exceeded that of spores cultured in PDB across all time points. At the 12 h mark, the germination rates of spores cultured in PDB and sterile water were nearly identical (Figure 3H), but the spore size was significantly smaller than that of spores cultured in sterile water. Considering the consumption of experimental materials and lowering the cost, the spores incubated in sterile water for 12 h (90.33% germination rate) were selected to observe their spore morphology.

The bacterial suspension exhibited the most pronounced antagonistic effect against *F. oxysporum* (Table 4). As with the results of Section 3.2.3, the antagonistic effect of the sterile filtrate was weak, which may be due to the fact that strain LK−1 produces fewer inhibitory substances or has a shorter lifespan.

Under standard culture conditions, the spores exhibit normal germination characterized by elongated bud tubes (Figures 3G,H). Following treatment with strain LK−1, the spores exhibit characteristics such as inflation (Figure 3J) and deformation (Figures 3I,K,L), which adversely impact their normal germination and contribute to antagonistic effects.

#### 3.2.5 The promoting effect of *Bacillus subtilis* on *Lycium barbarum*

The growth index of *L. barbarum* revealed that strain LK−1 significantly enhanced the growth of *L. barbarum* plants, primarily evidenced by increases in plant height, root length, aboveground fresh weight, underground dry weight, and leaf area. Compared with the blank control (CK), they increased by 37.25, 33.95, 93.02, 88.14, and 38.13%. There was a significant increase in the dry matter of *L. barbarum* roots after treatment with strain LK-1 (E). Under the stress of *F. oxysporum*, LK−1 was able to enhance the growth parameters of *L. barbarum* as demonstrated by the following results: compared to the pathogenic bacteria control group (P) treated with *F. oxysporum* alone, treatment (E + P) resulted in increases of 2.45% in plant height, 2.06% in root length, and significant enhancements of 70.49, 25.92, 52.97, 71.36, and 28.99% in aboveground fresh weight, belowground fresh weight, aboveground dry weight, belowground dry weight, and leaf area, respectively (Table 5).

**Table 5:** Effects of different treatments on growth indexes of *L. barbarum* seedlings.

Treatment	Plant height(cm)	Root length(cm)	Aboveground fresh weight(mg)	Underground fresh weight(mg)	Aboveground dry weight(mg)	Underground dry weight(mg)	Area of leaves(cm^2^)
P	2.45 ± 0.35ab	11.15 ± 2.43ab	120.13 ± 38.06c	18.4 ± 5.47ab	11.97 ± 3.24c	4.05 ± 1.21b	1.38 ± 0.38b
E + P	2.51 ± 0.66ab	11.38 ± 2.62ab	204.81 ± 41.61b	23.17 ± 5.48a	18.31 ± 3.52b	6.94 ± 1.25a	1.78 ± 0.37b
CK	2.04 ± 0.25b	8.69 ± 2.89b	158.56 ± 39.82bc	17.77 ± 10.84ab	14.59 ± 3.78bc	4.59 ± 2.95b	1.36 ± 0.33b
E	2.8 ± 0.66a	11.64 ± 2.32a	306.06 ± 70.79a	15.3 ± 4.25b	27.45 ± 6.84a	6.34 ± 2.39ab	2.26 ± 0.46a

P: treatment group of *F. oxysporum*; E + P: co-treatment group of LK-1 and *F. oxysporum*; CK: blank control group; E: treatment group of strain LK-1. The lowercase letters indicates the significant differences in *Lycium barbarum* under different treatments.

### 3.3 Single-factor test

**Figure 4:**
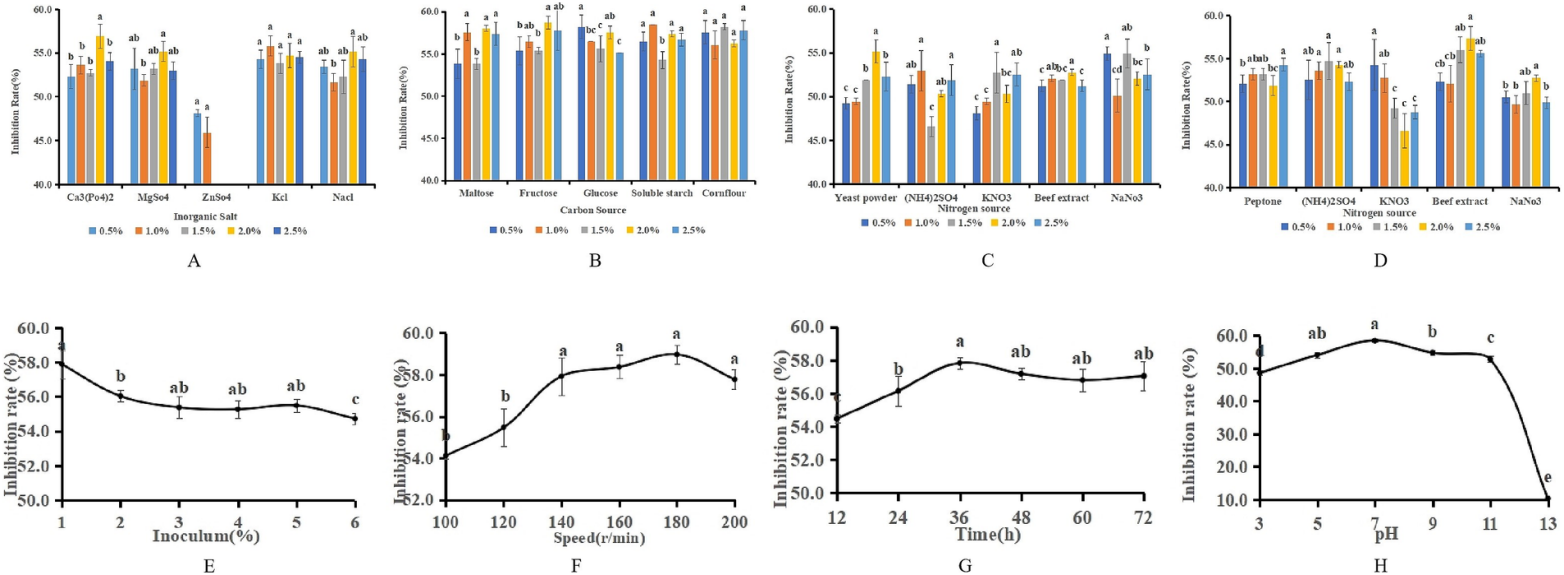
Optimization of fermentation media and conditions. **(A)** Replacement of inorganic salts. **(B)** Replacement of carbon sources. **(C)** Replacement of yeast powder. **(D)** Replacement of peptone. Data with different letters indicate a significant difference at the 0.05 level. **(E)** Amount of inoculation. **(F)** Speed of rotation. **(G)** Incubation time. **(H)** pH.

The single-factor analysis revealed that the most pronounced antagonistic effect, reaching 56.92%, was achieved when the inorganic salt in the fermentation medium was substituted with Ca_3_(PO4)_2_ at a concentration of 2%. When ZnSO_4_ was substituted for the inorganic salt, a negative correlation was observed between the antagonistic capacity of LK−1 and the concentration of ZnSO_4_; specifically, as the concentration increased, the antagonistic ability diminished. This suggests that elevated levels of ZnSO_4_ are detrimental to the growth of LK−1. Substituting peptone with beef paste at a concentration of 2% in the fermentation medium resulted in an antagonistic effect of 57.36%. When sucrose was utilized as the carbon source at a concentration of 2%, the fermentation broth exhibited the highest antagonistic activity, measuring 58.68% (Figure 4). In light of the findings from the single-factor analysis and cost considerations, the medium was initially substituted with 0.5% glucose, 2% beef paste, 2% NaCl, and 0.5% yeast powder.

**Figure 5:**
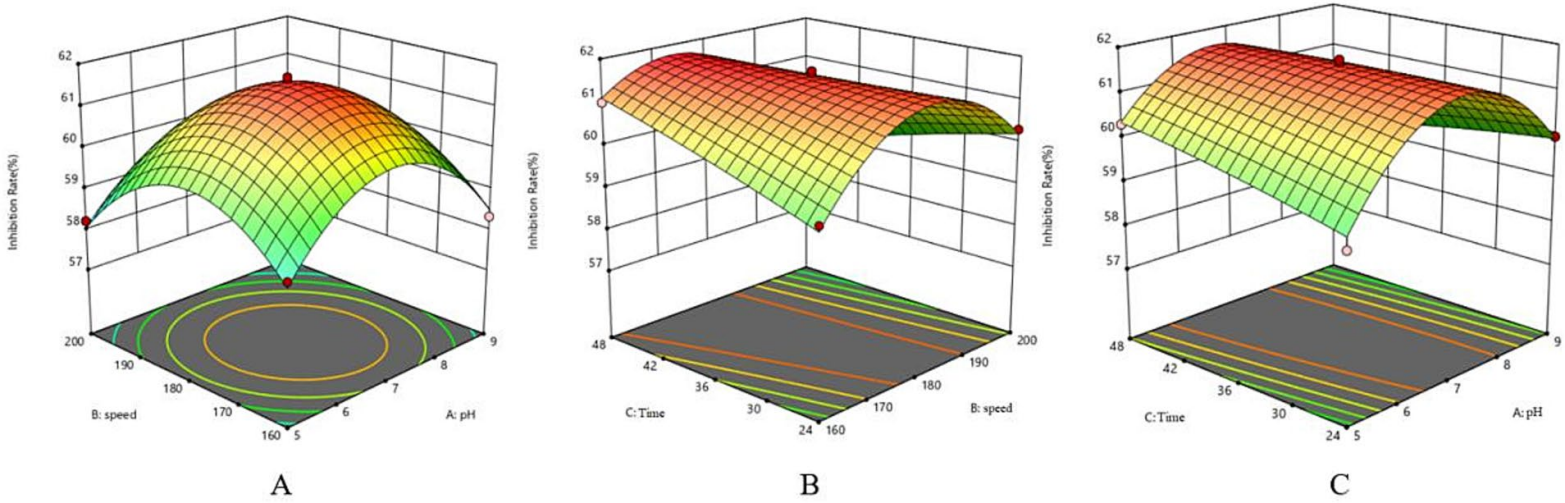
Response surface method surface map. **(A)** Surface map of speed and pH. **(B)** Surface map of incubation time and speed. **(C)** Surface map of incubation time and pH.

The single-factor test showed that when the fermentation time was 36 h, the antagonistic activity reached 57.82%; when the inoculum of bacteria received was 1%, the antagonistic activity reached 57.87%; when the pH was 7, the antagonistic activity reached 58.46%; and when the rotational speed was 180 rpm, the antagonistic activity reached 58.97% (Figure 5). The antagonistic activity was reduced when the fermentation conditions were higher or lower than this condition.

### 3.4 Response surface optimization

#### 3.4.1 Plackett–Burman

The influence of the selected factors on the rate of inhibition, ranked in descending order, is as follows: pH > speed>time>beef paste>glucose>NaCl>inoculum (Tables 6, 7).

**Table 6:** Design results of Plackett–Burman and experiments.

Treatment no.	Glucose	NaCl	Beef paste	Time	Inoculum	pH	Speed	Inhibition rate (%)
1	1	1	−1	1	1	1	−1	60.56
2	−1	1	1	−1	1	1	1	60.77
3	1	−1	1	1	−1	1	1	63.37
4	−1	1	−1	1	1	−1	1	57.92
5	−1	−1	1	−1	1	1	−1	57.76
6	−1	−1	−1	1	−1	1	1	60.48
7	1	−1	−1	−1	1	−1	1	56.85
8	1	1	−1	−1	−1	1	−1	56.77
9	1	1	1	−1	−1	−1	1	57.92
10	−1	1	1	1	−1	−1	−1	56.02
11	1	−1	1	1	1	−1	−1	55.61
12	−1	−1	−1	−1	−1	−1	−1	54.46

**Table 7:** Significance analysis and results of Plackett–Burman experiments.

Factor	F	*p*	Contribution rate	Order of importance
Glucose	0.86	0.41	1.47	5
NaCl	0.13	0.73	0.23	6
Beef paste	1.24	0.33	2.12	4
Time	5.68	0.08	9.72	3
Inoculum	0.01	0.91	0.02	7
pH	27.84	0.01	47.63	1
Speed	16.56	0.02	28.33	2

#### 3.4.2 Steepest climb test

The fermentation broth of LK−1 exhibited the highest inhibition rate in test 3 (Table 8). Therefore, the conditions established in test 3 (fermentation time of 36 h, pH 7, and rotational speed of 180 rpm) should be designated as the central point for the factor levels in the response surface methodology.

**Table 8:** Steepest climb test.

Treatment no.	Time (h)	pH	Speed (r/min)	Inhibition rate (%)
1	12	5	140	56.86
2	24	6	160	58.31
3	36	7	180	59.82
4	48	8	200	57.92
5	60	9	220	56.80

#### 3.4.3 Box–Behnken tests

Following the outcomes of the steepest ascent test, the Box–Behnken design principle from Design-Expert 12 software was employed to perform a three-factor, three-level experiment on the selected primary factors. Each experiment was replicated three times, and the results of this experimental design are presented in Table 9.

**Table 9:** Box–Behnken design and corresponding results.

Treatment no.	A	B	C	Inhibition rate (%)
1	5	160	36	58.68
2	9	160	36	58.33
3	5	200	36	58.22
4	9	200	36	57.29
5	5	180	24	59.03
6	9	180	24	59.95
7	5	180	48	60.3
8	9	180	48	59.38
9	7	160	24	59.61
10	7	200	24	60.3
11	7	160	48	61
12	7	200	48	58.68
13	7	180	36	61.57
14	7	180	36	61.57
15	7	180	36	61.65
16	7	180	36	61.69
17	7	180	36	61.34

The analysis of variance for the response surface regression model is presented in Table 8. The multivariate equation correlating the inhibition rate (Y) with pH (A), speed (B), and time (C) was derived from the results analyzed using Design-Expert 12 software, expressed as Y = 61.56–0.16A - 0.3912B + 0.0587C - 0.145AB - 0.46 AC - 0.7525 BC - 1.83A^2^-1.6B^2^-0.0657C^2^.

The regression model has an *F* = 62.54 and *p* < 0.00001 (Table 10), indicating that the model has reached a highly significant level. The coefficient of determination of the model, R^2^ = 0.9877 and R^2^adj = 0.9719, indicates that the error of the regression model is small, and the model is well fitted with a high degree of confidence.

**Table 10:** Analysis of variance for Box–Behnken experiment.

Source of variance	Square sum	Freedom	Mean square sum	F	*p*	Significance
mold	31.24	9	3.47	62.54	< 0.0001	**
A	0.2048	1	0.2048	3.69	0.0962	
B	1.22	1	1.22	22.06	0.0022	**
C	0.0276	1	0.0276	0.4975	0.5034	
AB	0.0841	1	0.0841	1.52	0.2581	
AC	0.8464	1	0.8464	15.25	0.0059	**
BC	2.27	1	2.27	40.81	0.0004	**
A^2^	14.15	1	14.15	254.94	< 0.0001	**
B^2^	10.79	1	10.79	194.37	< 0.0001	**
C^2^	0.0182	1	0.0182	0.3279	0.5848	
Residual	0.3885	7	0.0555			
Lost proposal	0.315	3	0.105	5.71	0.0627	
Pure terror	0.0735	4	0.0184			
Aggregate	31.63	16				

The symbol indicates the level of significance, that is, this factor has a significant effect on the response variable.

#### 3.4.4 Response surface optimization results and validation

Following response surface optimization, the optimal fermentation parameters for strain LK−1 were established as 0.5% glucose, 2% beef paste, 2% NaCl, 0.5% yeast powder, a fermentation duration of 46.261 h, an inoculum of 1%, a pH of 6.647, and a speed of 170.541 rpm. At the same time, in order to validate the accuracy and feasibility of the obtained optimal fermentation conditions, the fermentation conditions were adjusted to 0.5% glucose, 2% beef paste, 2% NaCl, 0.5% yeast powder, a fermentation time of 46 h, an inoculum of 1%, a pH of 6.6, and a speed of 170 rpm, and the experiment was carried out for three replications. The inhibition rate obtained was 62.5%. It was 1.43% higher than the theoretical value and 8.06% higher than the unoptimized value (54.44%). It showed that the fermentation conditions of strain LK−1 obtained by response surface optimization were accurate and reliable.

## 4 Conclusion and discussion

Wolfberry is reported to have benefits for human health. It has been used in traditional Chinese medicine for more than 2000 years. *Lycium barbarum* root rot causes a serious risk of yield loss and health hazards (Bodah, 2017). In the agriculture industry, *Bacillus* species are currently recognized as significant biological control agents against plant pathogens because of their fast colonization and ability to produce endospores (Shafi et al., 2017). Fungicides are extensively used to control many soil-borne diseases, but their effectiveness varies. Biological control using antagonistic microbes alone or as supplements to minimize the use of chemical pesticides in integrated plant disease management systems has become more critical in recent years. *Bacillus* species have attracted substantial attention as biocontrol agents for sustainable agriculture (Huang et al., 2022). Compared with chemical pesticides, the use of *Bacillus subtilis* LK−1 as a biocontrol agent has a smaller impact on non-target organisms and can better protect beneficial organisms, such as natural enemies and soil (Vasantha-Srinivasan et al., 2025).

*Bacillus subtilis* displays a wide range of biological functions through the production of a variety of antagonistic compounds. This tremendous versatility increases the industrial and environmental interest in *B. subtilis* strains, especially when considering their range of action against foodborne or phytopathogenic flora and their history of safe use in food. In this study, we identified a strain of *B. subtilis*, designated *B. subtilis* LK−1, through morphological examination, physiological and biochemical characterization, and molecular biology techniques. The preventive effects of this strain were evaluated, revealing its significant antagonistic activity against various *Fusarium* species via the secretion of bioactive compounds. They mainly contain three families: surfactins, iturins, and fengycins (Boka et al., 2016; Ma et al., 2016; Chen et al., 2017). It has been reported that the antimicrobial compounds of *Bacillus* species destroy mycelial structures and inhibit conidial germination for pathogenic fungi (Huang et al., 2022). They can promote plant growth by increasing nutrient availability and synthesizing plant hormones and volatile compounds (Xie et al., 2014). In addition to the direct antimicrobial effects, the antimicrobial compounds may also induce the plant’s own defense responses. When the antimicrobial substances secreted by LK-1 come into contact with the plant, they may activate the plant’s immune system and prompt the plant to produce more defense-related enzymes. Studies have shown that *Bacillus* species produce a variety of bacteriocins with antimicrobial activity, such as amylolysin, amylocyclicin, amysin, subtilin, subtilosin A, subtilosin B, and thuricin. They also produce important cyclic lipopeptides (CLPs), including iturins, fengicins, and surfactins, which can interact with the cell membranes of target pathogens to form pores and lead to an imbalance in transmembrane ion fluxes (Xie et al., 2021; Fu et al., 2014). In addition, *bacillus* species can produce a large number of hydrolytic enzymes, such as chitinases, chitosanases, glucanases, cellulases, lipases, and proteases. These compounds can efficiently hydrolyze the main components of fungal and bacterial cell walls and are involved in the suppression of plant pathogens (Chen et al., 2022). These defense responses may have a synergistic effect with the antimicrobial action described in this article. Future research could delve into the synergistic effects of different antagonistic actions on pathogenic fungi, thereby providing more precise evidence for optimizing biological control (Bonaterra et al., 2022).

The antibacterial compounds produced by strain LK-1 can induce various abnormalities in the mycelium and spores of *F. oxysporum*, thus inhibiting the spread and propagation of pathogenic fungi. In addition, compared to the *L. barbarum* seedlings in the CK, the plant height, root length, fresh weight above ground, underground fresh weight, dry weight above ground, underground dry weight, and leaf area indexes of *L. barbarum* seedlings treated with LK-1 fermentation broth (1 × 10^8^ cfu/mL) were all significantly increased, the same as in a previous study (Ganeshan et al., 2024). In addition, *Bacillus licheniformis* can induce the synthesis of plant growth hormones to promote plant growth (Gkorezis et al., 2016); *Bacillus amyloliquefaciens* can improve the soil ecological environment of tomato roots, loosen the soil, and make it conducive to tomato growth (Wang et al., 2022); *bacillus methylotrophic* can enhance the activity of substrate enzymes and the content of available nutrients to promote cucumber photosynthesis, accelerate the absorption, transportation, assimilation, and accumulation of nutrients, and thus promote cucumber growth. These studies also demonstrate the plant growth-promoting capabilities of *Bacillus* (Wang et al., 2023). In order to enhance the efficacy of *Bacillus* as a biological control agent, it is imperative to augment both its biomass and antibacterial compound production. In this study, the fermentation conditions of strain LK-1 were studied. The liquid fermentation process includes two aspects: one is the optimization of medium components, and the other is the optimization of fermentation conditions.

Different fermentation conditions can affect the activity of antibacterial substances to different degrees, and some even inhibit the secretion of antibacterial active substances. We used a single-factor test to screen the biocontrol effect of strain LK-1 as an indicator. It can be seen from the test that the antibacterial activity of the fermentation product of strain LK-1 is closely related to the fermentation conditions when the inorganic salt is ZnSO_4_; the higher the concentration, the lower the biocontrol effect. The increase in zinc sulfate concentration leads to a gradual decrease in biocontrol efficacy. This is likely because excessively high concentrations of zinc sulfate can inhibit the growth and metabolism of the bacterial strain, thereby affecting the synthesis and secretion of its antimicrobial compounds (Aveledo et al., 2018). Finally, we optimized the fermentation conditions using response surface methodology; optimal conditions included 0.5% glucose, 2% beef paste, 2% NaCl, 0.5% yeast powder, a time of 46 h, an inoculum of 1%, a pH of 6.6, and a speed of 170 rpm. Additionally, during the fermentation process, apart from the main antimicrobial compounds, other metabolic by-products may also be generated. These by-products may possess potential biological activities, such as antioxidant properties. Therefore, conducting a comprehensive identification and evaluation of these fermentation by-products may offer new ideas for the development of multifunctional biocontrol (Ricci et al., 2021).

## Data Availability

The datasets presented in this study can be found in online repositories. The names of the repository/repositories and accession number(s) can be found in the article/Supplementary material.

## References

[ref1] Aguirre A. M. Bassi A. (2013). Investigation of biomass concentration, lipid production, and cellulose content in *Chlorella vulgaris* cultures using response surface methodology. Biotechnol. Bioeng. 110, 2114–2122. doi: 10.1002/bit.24871, PMID: 23436332

[ref2] Allard-Massicotte R. Tessier L. Lecuyer F. Lakshmanan V. Lucier J. F. Garneau D.. (2016). *Bacillus subtilis* early colonization of *arabidopsis thaliana* roots involves multiple chemotaxis receptors. MBio 7, e1616–e1664. doi: 10.1128/mbio.01664-16, PMID: 27899502 PMC5137498

[ref3] Aveledo R. Aveledo A. Vázquez C. (2018). Study of bacterial sensitivity in zinc sulfate solutions by microcalorimetry. J. Therm. Anal. Calorim. 133, 773–777. doi: 10.1007/s10973-018-7000-x

[ref4] Aydi Ben Abdallah R. Jabnoun-Khiareddine H. Nefzi A. Mokni-Tlili S. Daami-Remadi M. (2016). Biocontrol of fusarium wilt and growth promotion of tomato plants using endophytic bacteria isolated from *solanum elaeagnifolium* stems. J. Phytopathol. 164, 811–824. doi: 10.1111/jph.12501

[ref5] Bai L. Li X. Cao Y. T. Song Z. Ma K. Fan Y.. (2020). Fusarium culmorum and fusarium equiseti causing root rot disease on *Lycium barbarum* (goji berry) in China. Plant Dis. 104:3066. doi: 10.1094/pdis-11-19-2313-pdn

[ref6] Beauregard P. B. Chai Y. Vlamakis H. Losick R. Kolter R. (2013). *Bacillus subtilis* biofilm induction by plant polysaccharides. Proc. Natl. Acad. Sci. USA 110, E1621–E1630. doi: 10.1073/pnas.1218984110, PMID: 23569226 PMC3637697

[ref7] Bhatia S. K. Mehta P. K. Bhatia R. K. Bhalla T. C. (2014). Optimization of arylacetonitrilase production from Alcaligenes sp. Mtcc 10675 and its application in mandelic acid synthesis. Appl. Microbiol. Biotechnol. 98, 83–94. doi: 10.1007/s00253-013-5288-9, PMID: 24104468

[ref8] Bodah E. T. (2017). Root rot diseases in plants: a review of common causal agents and management strategies. Agri. Res. Tech.: Open. Access. J. 5, 53–63. doi: 10.19080/artoaj.2017.05.555661

[ref9] Boka B. Manczinger L. Kecskemeti A. Chandrasekaran M. Kadaikunnan S. Alharbi N. S.. (2016). Ion trap mass spectrometry of surfactins produced by *Bacillus subtilis* szmc 6179j reveals novel fragmentation features of cyclic lipopeptides. Rapid Commun. Mass Spectrom. 30, 1581–1590. doi: 10.1002/rcm.7592, PMID: 27321846

[ref10] Bonaterra A. Badosa E. Daranas N. Francés J. Roselló G. Montesinos E. (2022). Bacteria as biological control agents of plant diseases. Microorganisms. 10:1759. doi: 10.3390/microorganisms10091759, PMID: 36144361 PMC9502092

[ref11] Caulier S. Nannan C. Gillis A. Licciardi F. Bragard C. Mahillon J. (2019). Overview of the antimicrobial compounds produced by members of the *Bacillus subtilis* group. Front. Microbiol. 10:302. doi: 10.3389/fmicb.2019.00302, PMID: 30873135 PMC6401651

[ref12] Chen Y. Liu S. A. Mou H. Ma Y. Li M. Hu X. (2017). Characterization of lipopeptide biosurfactants produced by *bacillus licheniformis* mb 01 from marine sediments. Front. Microbiol. 8:871. doi: 10.3389/fmicb.2017.00871, PMID: 28559889 PMC5432566

[ref13] Chen S. Wang J. Zhang B. Liu X. (2022). Efficacy of *Bacillus velezensis* on soybean root rot in pot experiments and detection of defense enzyme activity. Mol. Plant. Breed. 20, 6492–6500. doi: 10.13271/j.mpb.020.006492

[ref14] Dandan Z. Li S. Han X. Li C. Ni Y. Hao J. (2020). Physico-chemical properties and free amino acids profiles of six wolfberry cultivars in zhongning. J. Food Compos. Anal. 88:103460. doi: 10.1016/j.jfca.2020.103460, PMID: 40535901

[ref15] Dhandhukia P. C. Thakkar V. R. (2008). Response surface methodology to optimize the nutritional parameters for enhanced production of jasmonic acid by Lasiodiplodia theobromae. J. Appl. Microbiol. 105, 636–643. doi: 10.1111/j.1365-2672.2008.03803.x, PMID: 18397253

[ref16] Donato V. Ayala F. R. Cogliati S. Bauman C. Costa J. G. Lenini C.. (2017). *Bacillus subtilis* biofilm extends *caenorhabditis elegans* longevity through downregulation of the insulin-like signalling pathway. Nat. Commun. 8:14332. doi: 10.1038/ncomms14332, PMID: 28134244 PMC5290332

[ref17] Dong X. Cai M. (2001). Manual of systematic identification of common Bacteria. eds. Xiu-Zhu Dong and Miao-Ying Cai. Beijing: Sicence Press.

[ref18] Donno D. Beccaro G. L. Mellano M. G. Cerutti A. K. Bounous G. (2015). Goji berry fruit (Lycium spp.): antioxidant compound fingerprint and bioactivity evaluation. J. Funct. Foods 18, 1070–1085. doi: 10.1016/j.jff.2014.05.020

[ref19] Estrada R. Jr. Gudmestad N. C. Rivera V. V. Secor G. A. (2010). Fusarium graminearum as a dry rot pathogen of potato in the Usa: prevalence, comparison of host isolate aggressiveness and factors affecting aetiology. Plant Pathol. 59, 1114–1120. doi: 10.1111/j.1365-3059.2010.02343.x

[ref20] Fu W. Gao Y. Zhang X. (2014). Research Progress on Iturin. Anhui. Agril. Sci. Bull. 20, 23–26. doi: 10.16377/j.cnki.issn1007-7731.2014.24.022

[ref21] Ganeshan S. Settu V. Mannu J. Annaiyan S. Muthusamy G. Arun A.. (2024). Genomic analysis of *Bacillus subtilis* sub sp. subtilis geb5 reveals its genetic assets for nematicidal and plant growth promoting mechanisms. Rhizosphere-Neth. 31:100953. doi: 10.1016/j.rhisph.2024.100953, PMID: 40535901

[ref22] Gkorezis P. Van Hamme J. Bottos E. Thijs S. Balseiro-Romero M. Monterroso C.. (2016). Draft genome sequence of *Bacillus licheniformis* strain GB2, a hydrocarbon-degrading and plant growth-promoting soil bacterium. Genome Announc. 4, e00608–e00616. doi: 10.1128/genomeA.00608-16, PMID: 27340073 PMC4919412

[ref23] Hsieh S. Liu J. Pua X. Ting Y. Hsu R. Cheng K. (2016). Optimization of *Lactobacillus acidophilus* cultivation using taro waste and evaluation of its biological activity. Appl. Microbiol. Biotechnol. 100, 2629–2639. doi: 10.1007/s00253-015-7149-1, PMID: 26572522

[ref24] Hu X. Qu Y. Chu Q. Li W. He J. (2018). Investigation of the neuroprotective effects of *lycium barbarum* water extract in apoptotic cells and alzheimer's disease mice. Mol. Med. Rep. 17, 3599–3606. doi: 10.3892/mmr.2017.8310, PMID: 29257339 PMC5802160

[ref25] Huang Y. Zhang X. Xu H. Zhang F. Zhang X. Yan Y.. (2022). Isolation of lipopeptide antibiotics from *bacillus siamensis*: a potential biocontrol agent for fusarium graminearum. Can. J. Microbiol. 68, 403–411. doi: 10.1139/cjm-2021-0312, PMID: 35171710

[ref26] Inbaraj B. S. Lu H. Hung C. F. Wu W. B. Lin C. L. Chen B. H. (2008). Determination of carotenoids and their esters in fruits of *Lycium barbarum* linnaeus by hplc–dad–apci–ms. J. Pharm. Biomed. Anal. 47, 812–818. doi: 10.1016/j.jpba.2008.04.001, PMID: 18486400

[ref27] Jan F. Arshad H. Ahad M. Jamal A. Smith D. L. (2023). In vitro assessment of *Bacillus subtilis* fj3 affirms its biocontrol and plant growth promoting potential. Front. Plant Sci. 14:1205894. doi: 10.3389/fpls.2023.1205894, PMID: 37538061 PMC10395516

[ref28] Jiang C. Wu F. Yu Z. Xie P. Ke H. Li H.. (2015). Study on screening and antagonistic mechanisms of *bacillus amyloliquefaciens* 54 against bacterial fruit blotch (bfb) caused by *acidovorax avenae* subsp. Citrulli. Microbiol. Res. 170, 95–104. doi: 10.1016/j.micres.2014.08.009, PMID: 25267487

[ref29] Jiang C. Yao X. Mi D. Li Z. Yang B. Zheng Y.. (2019). Comparative transcriptome analysis reveals the biocontrol mechanism of *Bacillus velezensis* f21 against fusarium wilt on watermelon. Front. Microbiol. 10:652. doi: 10.3389/fmicb.2019.00652, PMID: 31001229 PMC6456681

[ref30] Kamil F. H. Saeed E. E. El-Tarabily K. A. AbuQamar S. F. (2018). Biological control of mango dieback disease caused by lasiodiplodia theobromae using streptomycete and non-streptomycete actinobacteria in the United Arab Emirates. Front. Microbiol. 9:829. doi: 10.3389/fmicb.2018.00829, PMID: 29780366 PMC5945903

[ref31] Kulaitienė J. Vaitkevičienė N. Jarienė E. Černiauskienė J. Jeznach M. Paulauskienė A. (2020). Concentrations of minerals, soluble solids, vitamin c, carotenoids and toxigenic elements in organic goji berries (*Lycium barbarum* l.) cultivated in Lithuania. Biol. Agric. Hortic. 36, 130–140. doi: 10.1080/01448765.2020.1748714

[ref32] Liu C. Ruan H. Shen H. Chen Q. Zhou B. Li Y.. (2007). Optimization of the fermentation medium for alpha-galactosidase production from aspergillus foetidus zu-g1 using response surface methodology. J. Food Sci. 72, M120–M125. doi: 10.1111/J.1750-3841.2007.00328.x, PMID: 17995779

[ref33] Ma Y. Kong Q. Qin C. Chen Y. Chen Y. Lv R.. (2016). Identification of lipopeptides in *Bacillus megaterium* by two-step ultrafiltration and lc-esi-ms/ms. AMB Express 6:79. doi: 10.1186/s13568-016-0252-6, PMID: 27639854 PMC5026979

[ref34] Maryani N. Sandoval-Denis M. Lombard L. Crous P. W. Kema G. H. J. (2019). New endemic fusarium species hitch-hiking with pathogenic fusarium strains causing Panama disease in small-holder banana plots in Indonesia. Pers.: Mol. Phylogeny Evol. Fungi. 43, 48–69. doi: 10.3767/persoonia.2019.43.02, PMID: 32214497 PMC7085855

[ref35] Naveed M. Tianying H. Wang F. Yin X. Chan M. W. H. Ullah A.. (2022). Isolation of lysozyme producing *bacillus subtilis* strains, identification of the new strain *bacillus subtilis* bsn314 with the highest enzyme production capacity and optimization of culture conditions for maximum lysozyme production. Curr. Res. Biotechnol. 4, 290–301. doi: 10.1016/j.crbiot.2022.06.002

[ref36] Patel M. Islam S. Husain F. M. Yadav V. K. Park H. Yadav K. K.. (2023). *Bacillus subtilis* er-08, a multifunctional plant growth-promoting rhizobacterium, promotes the growth of fenugreek (trigonella foenum-graecum l.) plants under salt and drought stress. Front. Microbiol. 14:1208743. doi: 10.3389/fmicb.2023.1208743, PMID: 37692403 PMC10483830

[ref37] Pieterse C. M. J. Zamioudis C. Berendsen R. L. Weller D. M. Van Wees S. C. M. Bakker P. A. H. M. (2014). Induced systemic resistance by beneficial microbes. Annu. Rev. Phytopathol. 52, 347–375. doi: 10.1146/annurev-phyto-082712-102340, PMID: 24906124

[ref38] Qin X. Wang X. Xu K. Yang X. Wang Q. Liu C.. (2022). Synergistic antitumor effects of polysaccharides and anthocyanins from lycium ruthenicum murr. on human colorectal carcinoma lovo cells and the molecular mechanism. Food. Sci. Nutr. 10, 2956–2968. doi: 10.1002/fsn3.2892, PMID: 36171788 PMC9469862

[ref39] Ricci A. Bertani G. Maoloni A. Bernini V. Levante A. Neviani E.. (2021). Antimicrobial activity of fermented vegetable byproduct extracts for food applications. Food Secur. 10:1092. doi: 10.3390/foods10051092, PMID: 34069051 PMC8156661

[ref40] Samaram S. Mirhosseini H. Tan C. P. Ghazali H. M. Bordbar S. Serjouie A. (2015). Optimisation of ultrasound-assisted extraction of oil from papaya seed by response surface methodology: oil recovery, radical scavenging antioxidant activity, and oxidation stability. Food Chem. 172, 7–17. doi: 10.1016/j.foodchem.2014.08.068, PMID: 25442517

[ref41] Samavati V. (2013). Central composite rotatable design for investigation of microwave-assisted extraction of okra pod hydrocolloid. Int. J. Biol. Macromol. 61, 142–149. doi: 10.1016/j.ijbiomac.2013.06.037, PMID: 23817104

[ref42] Senesi S. Celandroni F. Tavanti A. Ghelardi E. (2001). Molecular characterization and identification of *bacillus clausii* strains marketed for use in oral bacteriotherapy. Appl. Environ. Microbiol. 67, 834–839. doi: 10.1128/aem.67.2.834-839.2001, PMID: 11157251 PMC92655

[ref43] Shafi J. Tian H. Ji M. (2017). Bacillus species as versatile weapons for plant pathogens: a review. Biotechnol. Biotec. Eq. 31, 446–459. doi: 10.1080/13102818.2017.1286950

[ref44] Sharma S. Kumar S. (2024). Discovering fragile clades and causal sequences in phylogenomics by evolutionary sparse learning. Mol. Biol. Evol. 41:131. doi: 10.1093/molbev/msae131, PMID: 38916040 PMC11247346

[ref45] Tan S. Gu Y. Yang C. Dong Y. Mei X. Shen Q.. (2016). *Bacillus amyloliquefaciens* t-5 may prevent *ralstonia solanacearum* infection through competitive exclusion. Biol. Fertil. Soils 52, 341–351. doi: 10.1007/s00374-015-1079-z

[ref46] Tang X. He G. Chen Q. Zhang X. Ali M. A. M. (2004). Medium optimization for the production of thermal stable β-glucanase by *Bacillus subtilis* zjf-1a5 using response surface methodology. Bioresour. Technol. 93, 175–181. doi: 10.1016/j.biortech.2003.10.013, PMID: 15051079

[ref47] Tang Z. Sun D. Qian C. Chen Q. Duan S. Sun S. (2017). *Lycium barbarum* polysaccharide alleviates nonylphenol exposure induced testicular injury in juvenile zebrafish. Int. J. Biol. Macromol. 104, 618–623. doi: 10.1016/j.ijbiomac.2017.06.035, PMID: 28636878

[ref48] Tian X. Liang T. Liu Y. Ding G. Zhang F. Ma Z. (2019). Extraction, structural characterization, and biological functions of *Lycium barbarum* polysaccharides: a review. Biomol. Ther. 9:389. doi: 10.3390/biom9090389, PMID: 31438522 PMC6770593

[ref49] Uwaremwe C. Yue L. Liu Y. Tian Y. Zhao X. Wang Y.. (2021). Molecular identification and pathogenicity fusarium andalternaria species associated with root rot disease of wolfberry in Gansu and Ningxia provinces, China. Plant Pathol. 70, 397–406. doi: 10.1111/ppa.13285

[ref50] Vasantha-Srinivasan P. Park K. B. Kim K. Y. Jung W. J. Han Y. S. (2025). The role of Bacillus species in the management of plant-parasitic nematodes. Front. Microbiol. 15:1510036. doi: 10.3389/fmicb.2024.1510036, PMID: 39895938 PMC11782231

[ref51] Wan T. Zhao H. Wang W. (2018). Effects of the biocontrol agent Bacillus amyloliquefacien sn16-1 on the rhizosphere bacterial community and growth of tomato. J. Phytopathol. 166, 324–332. doi: 10.1111/jph.12690

[ref52] Wang Y. Jiang L. Liu J. Lu Q. Zheng Q. Guo Q.. (2020). Isolation and identification of *Bacillus subtilis* strain t-3 from soybean and its antagonism against severalcommonpathogenic fungi. IOP Conf. Ser.: earth. Environ. Sci. 546:052054. doi: 10.1088/1755-1315/546/5/052054

[ref53] Wang J. Ma F. Wang J. (2023). Effects of methylotrophic Bacillus on the growth of cucumber and the rhizosphere environment in substrate culture. Acta. Agric. Boreali-occident. Sin. 32, 899–909. doi: 10.7606/ji.ssn.1004-1389.2023.06.008

[ref54] Wang F. Qiu G. Luo W. (2022). Effects of *Bacillus amyloliquefaciens* B1619 on the growth promotion of processing tomato seedlings and the cultivation of strong seedlings. Xinjiang. Agric. Sci. 59, 1252–1259. doi: 10.6048/j.issn.1001-4330.2022.05.026

[ref55] Wei Y. Xu X. Tao H. Wang P. (2006). Growth performance and physiological response in the halophyte *lycium barbarum* grown at salt-affected soil. Ann. Appl. Biol. 149, 263–269. doi: 10.1111/j.1744-7348.2006.00092.x

[ref56] Xie Z. Luo Y. Zhang C. An W. Zhou J. Jin C.. (2023). Integrated metabolome and transcriptome during fruit development reveal metabolic differences and molecular basis between Lycium barbarum and Lycium ruthenicum. Meta 13, 3–14. doi: 10.3390/metabo13060680, PMID: 37367839 PMC10303592

[ref57] Xie S. Wu H. Zang H. Wu L. M. Zhu Q. Gao X. (2014). Plant growth promotion by spermidine-producing *bacillus subtilis* okb105. Mol. Plant-Microbe Interact. 27, 655–663. doi: 10.1094/mpmi-01-14-0010-r, PMID: 24678831

[ref58] Xie X. Zhang J. Wang H. Lei C. (2021). Research Progress on the synthesis and mechanism of action of natural Lipopeptide antibiotics in Bacillus. Chin. J. Antibiot. 46, 362–370. doi: 10.13461/j.cnki.cja.006979

[ref59] Yan Z. Reddy M. S. Ryu C. Mcinroy J. A. Wilson M. Kloepper J. W. (2002). Induced systemic protection against tomato late blight elicited by plant growth-promoting rhizobacteria. Phytopathology 92, 1329–1333. doi: 10.1094/phyto.2002.92.12.1329, PMID: 18943888

[ref60] Yang T. Hu Y. Yan Y. Zhou W. Chen G. Zeng X.. (2022). Characterization and evaluation of antioxidant and anti-inflammatory activities of flavonoids from the fruits of *Lycium barbarum*. Food Secur. 11:306. doi: 10.3390/foods11030306, PMID: 35159457 PMC8834156

[ref61] Zalila-Kolsi I. Kessentini S. Tounsi S. Jamoussi K. (2022). Optimization of *Bacillus amyloliquefaciens* blb 369 culture medium by response surface methodology for low cost production of antifungal activity. Microorganisms. 10:830. doi: 10.3390/microorganisms10040830, PMID: 35456879 PMC9029587

[ref62] Zhong K. Lin W. Wang Q. Zhou S. (2012). Extraction and radicals scavenging activity of polysaccharides with microwave extraction from mung bean hulls. Int. J. Biol. Macromol. 51, 612–617. doi: 10.1016/j.ijbiomac.2012.06.032, PMID: 22750575

[ref63] Zhou F. Jiang X. Wang T. Zhang B. Zhao H. (2018). *Lycium barbarum* polysaccharide (lbp): a novel prebiotics candidate for bifidobacterium and lactobacillus. Front. Microbiol. 9:1034. doi: 10.3389/fmicb.2018.01034, PMID: 29867910 PMC5968096

[ref64] Zhou Z. Xiao J. Fan H. Yu Y. He R. Feng X.. (2017). Polyphenols from wolfberry and their bioactivities. Food Chem. 214, 644–654. doi: 10.1016/j.foodchem.2016.07.105, PMID: 27507521

[ref65] Zhu S. Li X. Dang B. Wu F. Wang C. Lin C. (2022). *Lycium barbarum* polysaccharide protects hacat cells from pm2.5-induced apoptosis via inhibiting oxidative stress, er stress and autophagy. Redox Rep. 27, 32–44. doi: 10.1080/13510002.2022.2036507, PMID: 35130817 PMC8843200

[ref66] Zhu P. Zhao Y. Dai Z. Qin X. Yuan H. Jin Q.. (2020). Phenolic amides with immunomodulatory activity from the nonpolysaccharide fraction of *Lycium barbarum* fruits. J. Agric. Food Chem. 68, 3079–3087. doi: 10.1021/acs.jafc.9b07499, PMID: 32059104

