# Supplementary material for: Identification of antagonistic activity against Fusarium, and liquid fermentation of biocontrol Bacillus isolated from wolfberry (Lycium barbarum) rhizosphere soil
Source: Front Microbiol. 2025 Jul 15;16:1601945. doi: 10.3389/fmicb.2025.1601945 (PMC12303953; doi:10.3389/fmicb.2025.1601945)
Supplement: Supplementary file 1 [file Table_1.docx]

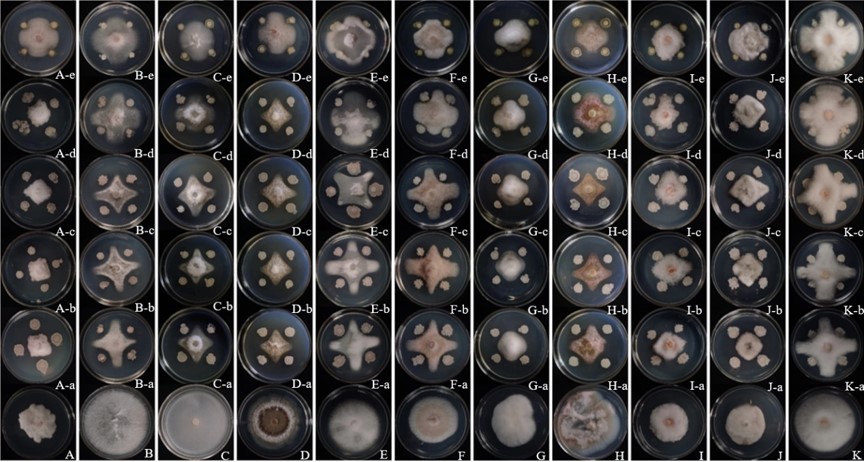


**Supplementary** **Figure 1. Effect of antagonism.** (A, C, D) *F. oxysporum* isolates isolated from *Astragalus* rhizosphere soil. (B) *F. oxysporum* isolates isolated from *L. barbarum* rhizosphere soil. (E-K) *F.oxysporum* isolates isolated from *Medicago sativa rhizosphere* soil. LK-1 has antagonistic effects on 11 species of Fusarium. LK-2 has antagonistic effects on 11 species of Fusarium. LK-3 haantagonistic effects on 11 species of Fusarium. LK-4 has antagonistic effects on 11 species of Fusarium. LK-5 has antagonistic effects on 11 species of Fusarium.
